# Autism Spectrum Disorder Risk Factor Met Regulates the Organization of Inhibitory Synapses

**DOI:** 10.3389/fnmol.2021.659856

**Published:** 2021-05-13

**Authors:** Pauline Jeckel, Martin Kriebel, Hansjürgen Volkmer

**Affiliations:** Department of Pharma and Biotech, NMI Natural and Medical Sciences Institute at the University of Tübingen, Reutlingen, Germany

**Keywords:** proto-oncogene Met, TSC2, gephyrin, synaptic connectivity, autism

## Abstract

A common hypothesis explains autism spectrum disorder (ASD) as a neurodevelopmental disorder linked to excitatory/inhibitory (E/I) imbalance in neuronal network connectivity. Mutation of genes including Met and downstream signaling components, e.g., PTEN, Tsc2 and, Rheb are involved in the control of synapse formation and stabilization and were all considered as risk genes for ASD. While the impact of Met on glutamatergic synapses was widely appreciated, its contribution to the stability of inhibitory, GABAergic synapses is poorly understood. The stabilization of GABAergic synapses depends on clustering of the postsynaptic scaffolding protein gephyrin. Here, we show *in vivo* and *in vitro* that Met is necessary and sufficient for the stabilization of GABAergic synapses *via* induction of gephyrin clustering. Likewise, we provide evidence for Met-dependent gephyrin clustering *via* activation of mTOR. Our results support the notion that deficient GABAergic signaling represents a pathomechanism for ASD.

## Introduction

Autism spectrum disorder (ASD) is a highly heterogeneous disease affecting ~2% of the population (Sharma et al., 2018). Major symptoms of ASD comprise restricted, repetitive behavior, behavioral rigidity, odd or intense interests, persistent deficits in social communication, interactions, and communicative behavior (Lai et al., 2014). The association with developmental deficits as a major cause qualified ASD as a neurodevelopmental disorder (Sahin and Sur, 2015). In this line, aberrant neurodevelopment in ASD was suggested to provoke an excitatory/inhibitory (E/I) imbalance of synaptic connectivity as an underlying pathomechanism (Nelson and Valakh, 2015).

E/I imbalance may be caused by deregulated expression or mutation of genes linked to excitatory and inhibitory synapse formation or stabilization. MET was identified as an ASD risk gene involved in synapse formation. Mutation of promoter sequences suggested reduced expression of MET and concomitant synaptic deficits (Peng et al., 2013). Accordingly, Met mutation in the developing rodent nervous system is linked to impaired dendritic complexity, spine morphology and, glutamatergic synapse formation (Qiu et al., 2014). Vice versa, Met ligand hepatocyte growth factor (HGF) is also involved in neuronal development and increases excitatory synapse densities in cortical neurons (Eagleson et al., 2016; Xie et al., 2016). Likewise, mutation of Tuberous Sclerosis Complex 2 (TSC2), a further autism risk gene, changes spine morphologies and glutamatergic transmission and points at a link between MET and TSC signaling and compromised neuronal connectivity in ASD (Tavazoie et al., 2005). Altogether, HGF activates Met and thereby induces intracellular signaling through the MAPK and the PI3K-Akt pathways (Trusolino et al., 2010). Both pathways may converge on Tsc1 and 2 which form an oligomeric complex to act as a negative regulator of mTOR (Laplante and Sabatini, 2012). As a consequence, mutation of TSC1 or TSC2 may provoke an increase in mTOR activity (Winden et al., 2018; Feliciano, 2020).

While considerable progress was achieved in the characterization of glutamatergic connectivity in ASD, less is known about a potential contribution of MET-dependent regulation of inhibitory transmission. Although mice deficient in Met activity showed decreased postsynaptic GABAergic inhibition (Lo et al., 2016), the precise role of Met to regulate the stability of inhibitory synapses is unknown.

The postsynaptic scaffold protein gephyrin is a crucial component for the stabilization of GABAergic synapses that localizes GABAA receptors to the postsynaptic membrane (Kneussel et al., 1999; Tyagarajan and Fritschy, 2014; Groeneweg et al., 2018). Loss of gephyrin expression in the postsynapse coincides with a destabilization of GABAergic presynaptic terminals. We have previously shown that gephyrin clustering and stabilization of GABAergic synapses is controlled by growth factor receptor signaling through mTOR activation including Fgfr1, Ntrk2, and EphA7 (Kriebel et al., 2011; Wuchter et al., 2012; Beuter et al., 2016). We here add the autism risk factor Met as a further receptor tyrosine kinase to regulate gephyrin clustering. We focused on the dentate gyrus as a regulator of the stress response (Fa et al., 2014) since ASD was reported to coincide with an increased stress response (Rumball et al., 2020). We provide evidence for a link to mTOR signaling and the contribution of the mTOR regulator Tsc2.

## Materials and Methods

### Lentiviral Vector Construction and Lentivirus Amplification

The construction of the lentiviral vector pLenti04C/SEW was described previously (Kriebel et al., 2020). miRNAs either serving as a control (miCTR) or for the knockdown of Met (miUTR, mi3923) and TSC2 (mi500, mi2675), respectively, were transferred into pLenti04C/SEW *via* Gateway^®^-recombination (Life Technologies GmbH, Darmstadt, Germany). Chosen target sequences for Met were ACTGTCCAGACGCCTTGTATG (mi3923), CCAGCCCACTTCCAAGAAACA (miUTR), for Tsc2 GGATGGATGTTGGCTTGTCCT (mi500), and TCATAGCCATGTGGTTCATTA (mi2675). Tsc2 knockdown vectors were named Vir1_Tsc2 (mi2675) or Vir2_Tsc2 (mi500) in a previous communication (Joly et al., submitted). The lentiviral particles were produced by lipofection of HEK239FT with lentiviral expression vectors and packaging plasmids pLP1, pLP2 and pLP/VSVG (Thermo Fisher Scientific, Waltham, MA, USA) according to the manufacturer’s protocols (viral titers approximately 5 × 10^7^ transducing units/ml as measured by Lenti-X p24 ELISA, Takara Bio Europe, Saint-Germain-en-Laye, France).

### Hippocampal Cultures

Primary cortical and hippocampal neurons were prepared from E17 rat embryos of either sex as described previously (Burkarth et al., 2007). Cells were seeded in NMEM with B27 supplement (Thermo Fisher Scientific, Waltham, MA, USA)/5% horse serum on polyethyleneimine (PEI)-coated 96-well Screenstar plates (Greiner, Nürtingen, Germany) at a density of 3.5 × 10^4^ cells/well. Four hours after plating, the medium was changed for serum-free MEM with B27 supplement.

### Transfection and Treatments of Primary Neurons

For transient transfection of hippocampal neurons, 300 ng of plasmid DNA were transfected using 1 μl of Lipofectamine 2000 (Thermo Fisher Scientific, Waltham, MA, USA) in 100 μl of the plating medium. Neurons were transfected at DIV 8 and were fixed after two additional days of incubation. Plasmids used were as follows: pEGFP-N1 (Takara Bio, Mountain View, CA, USA), mCherry-Rab5CA (Q79L; Addgene, #35138), mCherry-Rab5DN (S34N; Addgene, #35139), mRFP-Rab5 (Addgene, #14437), GFP-rab7 WT (Addgene, #12605), GFP-rab7 DN (Addgene, #12660), EGFP-Rab7A Q67L (Addgene, #28049), EGFP-Rab4A (Addgene, #49434), EGFP-Rab4AQ67L (Addgene, #49475), EGFP-Rab4AS22N (Addgene, #49476), HA-Rab11-DN (S25N), (Addgene, #101046), HA-Rab11-WT (Addgene, #101047), EGFP-Rab11AQ70L (Addgene, #49553), HA-Rab8a-WT (Addgene, #101048), HA-Rab8a-DN (T22N; Addgene, #101049), and HA-Rab8a-CA (Q67L) (Addgene, #101050). Inhibitors were dissolved in dimethylsulfoxide (DMSO), HGF (Merck KGaA, Darmstadt, Germany) in PBS and added to hippocampal neurons (DIV 9) to final concentrations of 50 ng/ml for HGF, 200 nm for mTOR inhibitor rapamycin (Merck), and 1 μM PHA-665752 (Merck KGaA, Darmstadt, Germany). Analyses were performed 30 min and 24 h (HGF Stimulation) or 24 h (Rapamycin treatment) after the addition of the reagents.

### Animals, Stereotactic Surgery, Transcardial Perfusion

For histological analyses, adult female Sprague–Dawley rats (250 g at the time point of surgery) were supplied by Janvier Labs (Saint-Berthevin Cedex, France). The housing of animals and submission to surgical procedures were performed in accordance with the European Union recommendations for the care and use of laboratory animals (2010/63/EU) and were approved by the regional authority (Regierungspräsidium Tübingen).

For stereotaxic injection of lentiviral suspensions, animals were deeply anesthetized with 2–5% isuflurane/oxygen, followed by an s.c. injection of metamizole (50 mg/kg). Stereotaxic injections into the dorsal dentate gyrus (AP: −2.9 mm, ML: ±2.5 mm, DV: −4.3 mm; all coordinates relative to Bregma) were performed using a Lab StandardTM Stereotaxic Instrument (Stoelting Company, Wood Dale, IL, USA) connected to a 701 RN Hamilton syringe (10 μl, 30 gauge, pst4; CS-Chromatographie Service GmbH, Langerwehe, Germany). For postoperative analgesia, 2 mg/kg meloxicam was administered by the s.c. injection following the surgical procedure. For fixation of brain tissue, animals were deeply anesthetized with ketamine (100 mg/kg i.p.) and xylazine (10 mg/kg i.p.) and transcardially perfused with 200 ml of PBS followed by 200 ml of 4% paraformaldehyde/PBS. Collected brain tissue was additionally fixed in 4% paraformaldehyde/PBS at 4°C for 60 min.

### Immunohistochemistry and Immunocytochemistry

Perfusion-fixed brains were washed in PBS and cut into 50 μm slices using a vibrating microtome (Vibratome VT1000S, Leica Microsystems, Wetzlar, Germany). After permeabilization (0.5% Triton X-100), slices were blocked by 1× BMB blocking reagent (Roche, Mannheim, Germany) for 2 h at room temperature. Further processing of brain slices and immunocytochemical staining was performed as described, previously (Beuter et al., 2016). Specimens were mounted on microscopic slides using Dako Fluorescent Mounting medium (Dako GmbH, Hamburg, Germany). Cultured neurons were fixed with 4% of paraformaldehyde/PBS for 20 min and permeabilized prior to immunostaining. Nuclei were stained using Hoechst Dye 33258 (1:1,000 in PBS; Merck KGaA, Darmstadt, Germany). The following primary antibodies were used: rabbit anti-Met (Abcam, Cambridge, UK, Cat. No. ab51067), Tuberin/TSC2 (D93F12; Cell Signaling Technology, Danvers, MA, Cat. No. 4308), monoclonal mouse anti-gephyrin (Synaptic Systems, Goettingen, Germany, Cat. No. 147021), polyclonal chicken anti-MAP2 (Thermo Fisher Scientific, Waltham, MA, USA, Cat. No. PA1-10005), polyclonal rabbit anti-PSD95 (Abcam, Cambridge, UK, Cat. No. ab18258), monoclonal rabbit anti-PSD95 (Synaptic Systems, Goettingen, Germany, Cat. No. 124011), polyclonal rabbit anti-VGAT (Synaptic Systems, Goettingen, Germany, Cat. No. 131003), monoclonal mouse anti-VGluT (Synaptic Systems, Goettingen, Germany, Cat. No. 135511), and polyclonal Phospho-p70 S6 Kinase (Thr389; Cell Signaling Technology, Danvers, MA, USA, Cat. No. 9205).

### Image Acquisition and Analysis

Confocal fluorescence images were acquired using a spinning disc confocal microscope (Cell Observer SD, Carl Zeiss Microscopy GmbH, Oberkochen, Germany) equipped with a 63× Plan-Apochromat oil immersion objective. Excitation and emission settings were kept constant for the recording of samples prepared from different experimental groups.

### Determination of p70 S6Kinase Phosphorylation and Protein Expression of Tsc2 (*in vitro*) and Met (*in vitro/in vivo*)

Protein expression levels of p70 S6Kinase, Tsc2 (*in vitro*), and Met were quantified by quantitative imaging. Confocal images of lentivirally transduced EGFP-positive neurons either in culture *in vitro* or in hippocampal slices of stereotaxically injected rats *in vivo* were further processed using ZEN 2 (Carl Zeiss Microscopy GmbH, Oberkochen, Germany). For each neuronal, EGFP-positive soma, a region of interest (ROI) of maximum diameter was selected from all confocal *z*-planes. Within each ROI, mean fluorescence intensities were quantified with the help of the ZEN measure function.

### Quantification of Synaptic Marker Expression

Cluster densities of synaptic proteins in the immune-stained specimen were quantified with the help of IMARIS software (Bitplane, Zurich, Switzerland). Confocal *z*-stacks of images taken from each sample were reconstructed in 3D. Twenty-five-micrometer segments of proximal dendrites, identified either by MAP2 immunoreactivity (primary neurons transduced with lentiviral suspensions or treated with natural ligands and synthetic molecules) or EGFP expression (hippocampal tissue, transfected primary neurons), were defined as ROIs using the IMARIS surface creation tool. ROIs also defined a mask, which served for the spatial detection and quantification of fluorescent signals indicative for immunostained synaptic marker proteins with the help of the IMARIS spot detection function. The range of gephyrin clusters localized to 25 μm of a proximal dendrite was approximately 10–25 with the number of respective segments indicated as “n” in the Figure legends.

### Statistic Evaluation

Statistical analyses were performed with GraphPad Prism 8.0.1. The *p*-values were assigned as follows: *p < 0.05; **p < 0.01; ***p < 0.001. For all groups, data were tested for Gaussian distribution using the D’Agostino and Pearson test. Each experiment was repeated at least three times. Within bar charts, error bars indicate the standard error of the mean (SEM).

## Results

### Gephyrin Co-localizes With Met

**Figure 1:**
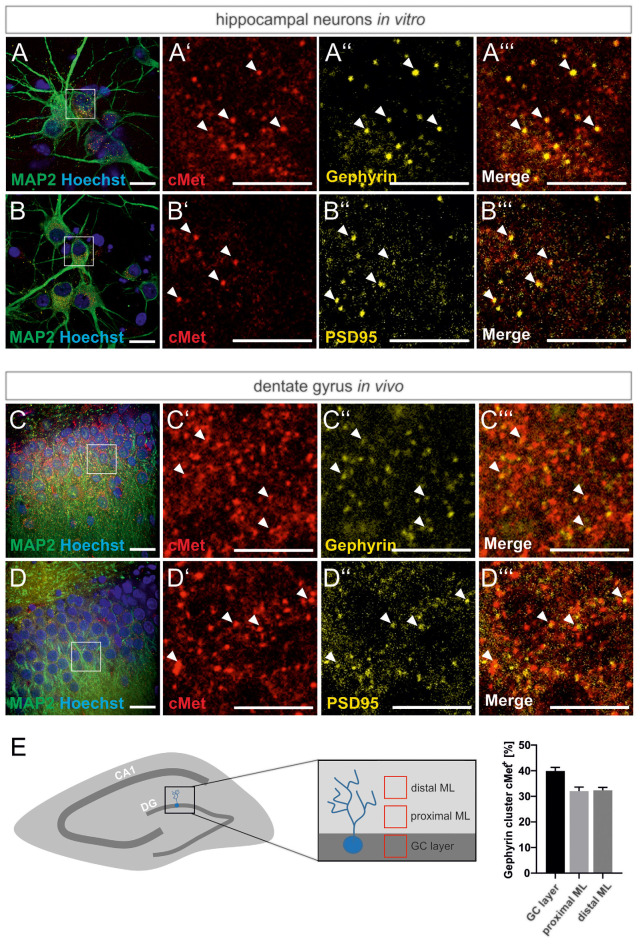
Met colocalizes with gephyrin and PSD95. Slices prepared from the dentate gyrus or hippocampal neurons were stained for the nuclear stain Hoechst, Met, gephyrin and PSD95 and submitted to confocal fluorescence microscopy. For overview, images of hippocampal neurons **(A,B)** or the granule cell layer of dentate gyrus **(C,D)** were stained with antibodies for MAP2 and Hoechst. Scale bar 20 μm. Insets in panels **(A–D)** are magnified in **(A’–A”’,B’–B”’,C’–C”’,D’–D”’)**. Scale bar 10 μm. **(A’–D’)**: Met staining, **(A”–D”)**: gephyrin or PSD95 staining as indicated,** (A”’–D”’)**: merged images of the respective representations in **(A’–A”,B’–B”,C’–C”,D’–D”)**. Arrow heads indicate colocalizing spots at the corresponding locations. **(E)** 20 μm^3^ cubes representative of the granule cell layer (GC layer), proximal (proximal ML) and distal molecular layer (distal ML) of the dentate gyrus were analyzed for the percentage of gephyrin clusters colocalizing with Met. *n* = 11 for all groups. Error bars: SEM.

For a link of the autism risk gene MET to the reorganization of GABAergic synapses, we first examined the colocalization of Met protein with the inhibitory synaptic marker gephyrin in primary hippocampal cultures and rat brain tissue. Neuronal cultures and hippocampal brain slices from adult rats, respectively, were stained for Met and gephyrin. The postsynaptic scaffold protein gephyrin is required for the clustering of GABA_A_ receptors in the postsynaptic membrane and serves as a marker for inhibitory synapses. As shown in Figure 1, colocalization of Met and gephyrin was observed in primary cultures and in the DG of brain slices (Figures 1A–A”’,C–C”’). In accordance with previous reports, we also found colocalization of c-Met and the postsynaptic marker of excitatory synapses, PSD95 (Figures 1B–B”’,D–D”’; Eagleson et al., 2013). A detailed analysis of gephyrin and Met colocalization in the DG revealed that 32–40% of gephyrin clusters were positive for Met (Figure 1E). Colocalization with gephyrin suggests a potential link between Met signaling and the organization of GABAergic synapses through the regulation of gephyrin clustering.

### Met Expression Is Required for Gephyrin Clustering

To assess the contribution of Met to gephyrin clustering, Met-specific miRNA expression cassettes were integrated into previously published lentiviral backbones (Kriebel et al., 2020). A knockdown strategy was chosen since reduced Met expression due to mutated promoter regions were shown to account for ASD and altered circuitry in humans (Rudie et al., 2012). The vectors drive the expression of Met-specific miRNA under the control of the CamKII promoter. Thus, miRNA expression is confined to glutamatergic principal neurons and excludes expression in hippocampal interneurons. Likewise, an additional synapsin promoter-EGFP expression cassette allows for the detection of infected neurons. Two independent miRNA constructs were cloned targeting the coding region (mi3923) or the 3’-untranslated region (UTR) of Met mRNA. A lentiviral vector expressing an miRNA without known target sequences for rat mRNAs served as a control. RT-PCR showed that both knockdown constructs reduced Met mRNA levels in neurons while both constructs also effectively downregulated Met protein expression as examined by quantitative immunofluorescence imaging *in vitro* (Supplementary Figure 1).

**Figure 2:**
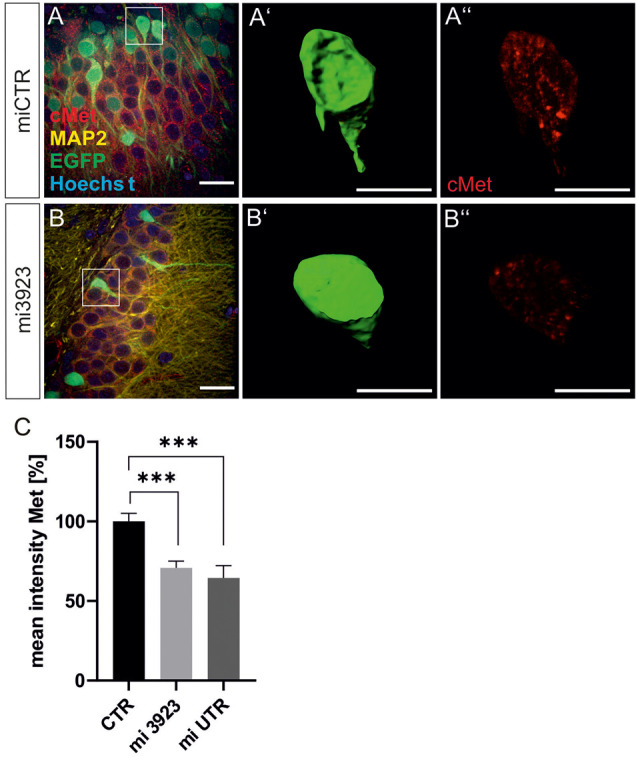
Lentiviral knockdown vectors decrease Met protein expression *in vivo*. Three lentivirus, expressing ineffective control miRNA (CTR) or targeting Met (mi3923 or miUTR) were injected into the dentate gyrus of 3 months old rats. Infected cells were visualized by coexpressed EGFP **(A,B)** in gephyrin-, MAP2, and Hoechst-stained slice preparations. Scale bar 20 μm. Insets in **(A,B)** were magnified in **(A’,B’,A”,B”)**. Scale bar 10 μm. The EGFP signals in single cells in **(A,B)** (see insets for example cells) were used to create a mask **(A’,B’)**, which served for the quantification of Met signals **(A”,B”)**. **(C)** Quantification of Met staining after lentiviral knockdown. miCTR *n* = Kruskal–Wallis test *H*_(2)_ = 24,6; miCTR (*n* = 78), mi3923 (*n* = 99), miUTR (*n* = 37). Error bars: SEM, ***p < 0.0001.

**Figure 3:**
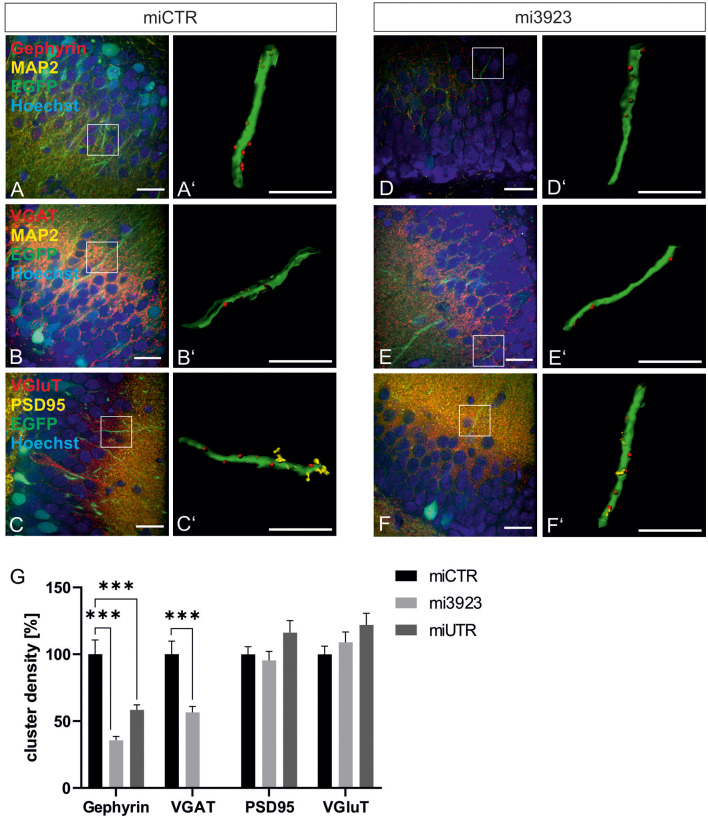
GABAergic synaptic markers are specifically lost after Met knockdown. Rat dentate gyrus were injected with lentivirus expressing either control (miCTR) or Met-specific miRNAs (mi3923, miUTR). **(A–C)** Images of miCTR transduced dendritic segments of dentate gyrus granular cells. (**D–F**) mi3923-transduced dendritic segments of dentate gyrus granular cells. Insets in **(A–F)** are enlarged in **(A’–F’)**. **(A’,D’)** Gephyrin spots, **(B’,E’)** VGAT spots, **(C’,F’)** PSD95/VGluT spots, each detected on EGFP-positive dendrites. Scale bars **(A–F)**: 20 μm, **(A’–F’)**: 10 μm. **(G)** Quantification of cluster densities of synaptic markers as measured on 25 μm dendritic segments. Gephyrin: miCTR *n* = 63, mi3923 *n* = 94, miUTR *n* = 56. Kruskal–Wallis test, *H*_(2)_ = 54.51. VGaT: miCTR *n* = 48, mi3923 *n* = 60. Mann–Whitney test. PSD95: miCTR *n* = 87, mi3923 *n* = 97, miUTR *n* = 63. Kruskal–Wallis test, *H*_(2)_ = 3.865. VGluT: miCTR *n* = 85, mi3923 *n* = 47, miUTR *n* = 61. Kruskal–Wallis test, *H*_(2)_ = 3.462. ***p < 0.0001. Error bars: SEM.

Lentiviral suspensions were injected in the dorsal dentate gyrus of rats *via* stereotaxic injection (for control of injection sites see Supplementary Figure 2). Two weeks after injection, animals were sacrificed for slice preparation and immunohistochemical analysis. Met expression was determined on cell bodies by quantitative immunostaining *in vivo* (Figure 2). In comparison to miCTR, c-Met-specific miRNAs reduced c-Met intensities to 70.8 and 64.5% (mi 3923, miUTR). Analysis of synaptic marker proteins revealed that knockdown of Met by mi3923 (35.7%) and miUTR (58.4%) reduced postsynaptic gephyrin cluster densities on proximal dendritic segments, significantly (Figures 3A–G). Application of two independent miRNA sequences excluded potential off-target effects. Presynaptic VGAT cluster density was reduced to 56.5%, indicating that Met expression is required for efficient gephyrin clustering and stabilization of GABAergic synapses. In contrast, PSD95 and VGluT cluster densities, indicative of glutamatergic post- and presynaptic compartments, respectively, remained unaffected implying that morphological synaptic alterations were specific for GABAergic synapses. The results show a role for the ASD risk factor Met in the stabilization of postsynaptic GABAergic synapses.

### Met Signaling Is Involved in Gephyrin Clustering

**Figure 4:**
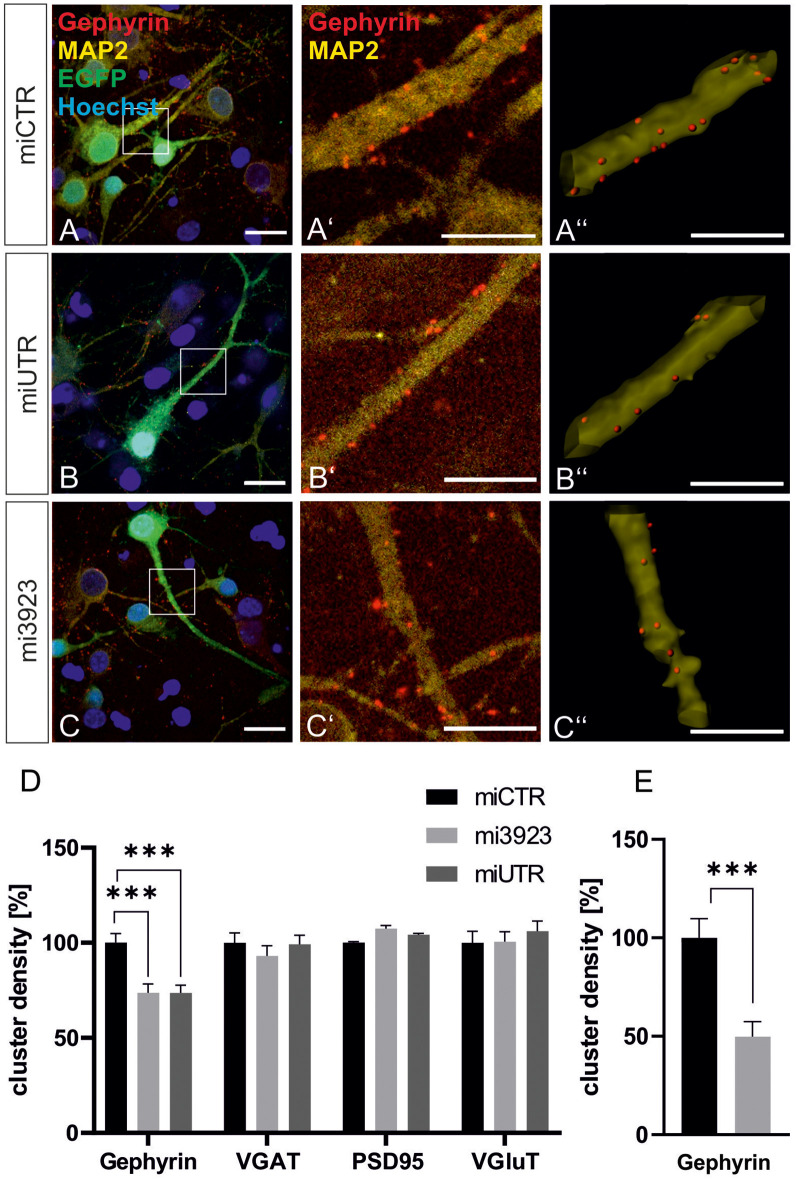
Met expression is required for gephyrin clustering *in vitro*. **(A–C)** Hippocampal neurons were infected with lentiviral vectors expressing miCTR **(A)**, or the Met knockdown constructs mi3923 **(B)** or miUTR **(C)**. Scale bar 20 μm. Insets in **(A–C)** are enlarged in the corresponding images **(A’–C’,A”–C”)**. Scale bar 10 μm. Panels **(A’–C’)** show confocal images depicting gephyrin (red) and the neuronal marker MAP2 (yellow). Panels **(A”–C”)** Gephyrin spots detected on dendritic segments used as a mask for the identification of gephyrin spots. **(D)** Quantification of synaptic marker densities on dendritic segments of 25 μm in length. One-way ANOVA and Tukey’s *post hoc* test. Gephyrin: miCTR *n* = 69, mi3923 *n* = 80, miUTR *n* = 71. *F*_(2,608)_ = 1.895. VGaT: miCTR *n* = 45, mi3923 *n* = 41, miUTR = 44. PSD95: miCTR *n* = 45, mi3923 *n* = 45, miUTR *n* = 45. VGluT: miCTR *n* = 46, mi3923 *n* = 44, miUTR *n* = 45. One-way ANOVA and Dunnett’s *post hoc* test. **(E)** Quantification of gephyrin cluster densities on cell somata. miCTR *n* = 35, mi3923 *n* = 30. Mann–Whitney test. ***p < 0.0001. Error bars: SEM.

To confirm data obtained in experiments *in vivo*, we analyzed the contribution of Met to gephyrin clustering in primary neurons, *in vitro*. Lentiviral Met knockdown vectors were applied to cultured hippocampal neurons (Figure 4). Both Met-specific miRNAs significantly reduced gephyrin cluster densities on proximal dendritic segments of hippocampal neurons to 73% as compared to miCTR (Figures 4A–D). On granule cell somata gephyrin cluster density was reduced to ~50% after Met knockdown (Figure 4E).

*In vitro*, presynaptic GABAergic terminals as well as pre-and postsynaptic glutamatergic markers quantified by VGAT, VGluT, and PSD95 staining, respectively, remained unaffected. In conclusion, the *in vitro* knockdown experiments confirm that Met expression is necessary for gephyrin clustering.

**Figure 5:**
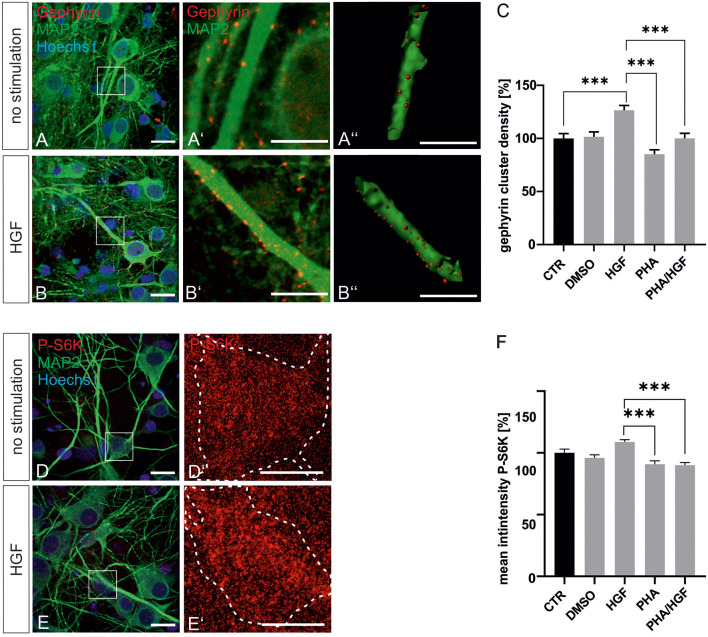
Hepatocyte growth factor (HGF) increases gephyrin clustering *in vitro*. Hippocampal neurons were left untreated or were treated with DMSO, HGF, or PHA-665752. Representative images of gephyrin- or MAP2-stained hippocampal neurons are shown in the absence **(A–A”)** or presence **(B–B”)** of HGF. Overview images are shown in (**A,B**; scale bar 20 μm) while insets are enlarged in (**A’,A”,B’,B”**; scale bar 10 μm). Panels **(A’,B’)** depict confocal images, **(A”,B”)** processed images for the quantification of gephyrin cluster (red) densities within masks of MAP2-positive dendritic segments (25 μm). (**C)** Quantification of gephyrin cluster densities. One-way ANOVA and Tukey’s multiple comparison test *F*_(4,364)_ = 0.7245. CTR: *n* = 75, HGF: *n* = 71, DMSO *n* = 78, PHA *n* = 73, PHA/HGF *n* = 72. ***p < 0.0001, error bars SEM. **(D,E)** Untreated hippocampal neurons **(D)** or treated with HGF **(E)** were stained for phosphorylated p70S6K (pS6K, scale bar 20 μm). Insets are enlarged in (**D’,E’**; scale bar 10 μm) for determination of mean fluorescence intensities of phosphorylated p70S6K signals within a mask of MAP2-positive cell bodies. Dashed outlines mark the area of the cell bodies. **(F)** Quantification of phosphorylated p70S6K intensities. CTR *n* = 57, DMSO *n* = 83, HGF *n* = 83, PHA *n* = 68, PHA/HGF *n* = 77. One-way ANOVA and Tukey’s multiple comparison test *F*_(4,363)_ = 3.369. ***p < 0.0001, error bars: SEM.

**Figure 6:**
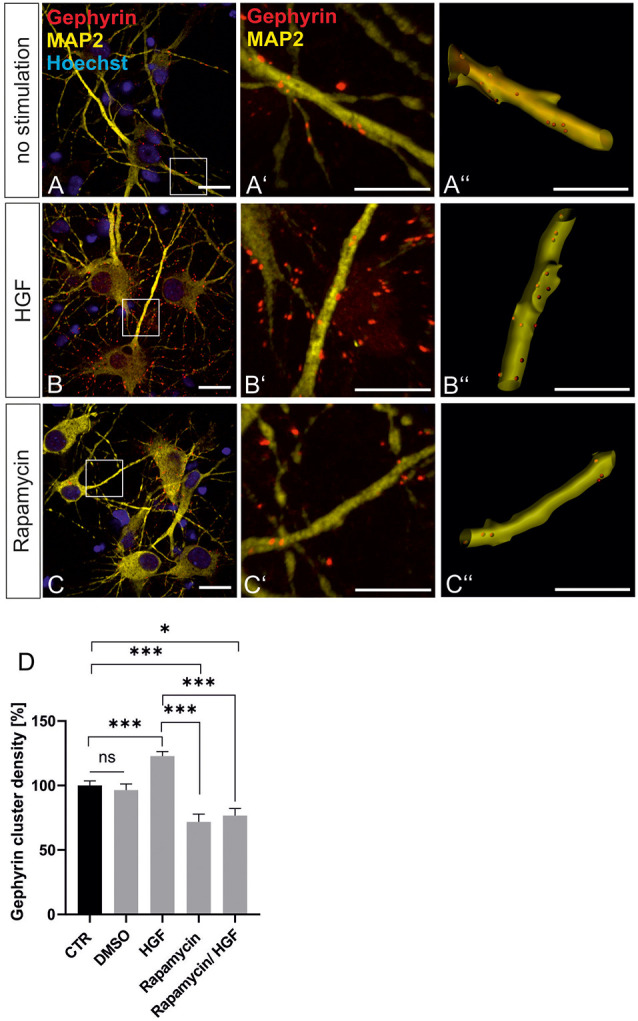
HGF-induced gephyrin clustering depends on mTOR activation. **(A–C)** Hippocampal neurons left untreated **(A)**, HGF stimulated **(B)** or HGF/rapamycin treated **(C)**. Scale bar 20 μm. Insets in **(A–C)** are enlarged in the corresponding images **(A’–C,A”–C”)**. Scale bar 10 μm. Panels **(A–C)** show confocal images depicting gephyrin (red), the neuronal marker MAP2 (yellow) and Hoechst stained nuclei (blue). **(A”–C”)** Gephyrin spots detected on dendritic segments used as a mask for the identification of gephyrin spots. **(D)** Quantification of synaptic marker densities on dendritic segments of 25 μm in length. Kruskal–Wallis test *H*_(4)_ = 69,32. Control *n* = 120, DMSO *n* = 43, HGF *n* = 111, rapamycin *n* = 45, HGF/rapamycin *n* = 43. *p = 0.203, ***p < 0.0001. Error bars: SEM. ns, not significant.

For further evidence of a contribution of Met to gephyrin clustering, we applied Met ligand HGF to induce gephyrin clustering in hippocampal neurons (Figures 5A–B”, quantified in **C**). Relative to untreated controls (100%), HGF treatment increased gephyrin clustering by 26% suggesting that c-Met activation is sufficient for increased gephyrin clustering. Application of the c-Met inhibitor PHA reduced HGF-induced gephyrin clustering to control levels indicating that Met activity is required for HGF-dependent gephyrin clustering. Likewise, HGF treatment of hippocampal neurons induced S6 kinase phosphorylation, which is indicative for mTOR activation after HGF treatment (Figures 5D–E’, quantified in **F**). For further confirmation, the mTOR-specific inhibitor rapamycin was applied (Figure 6). HGF-induced gephyrin cluster density was reduced to baseline levels implying that mTOR activation is required for gephyrin clustering. These findings support the hypothesis that Met and downstream mTOR activation account for HGF-induced gephyrin clustering.

### Late Endosomal Compartments Contribute to Gephyrin Clustering

**Figure 7:**
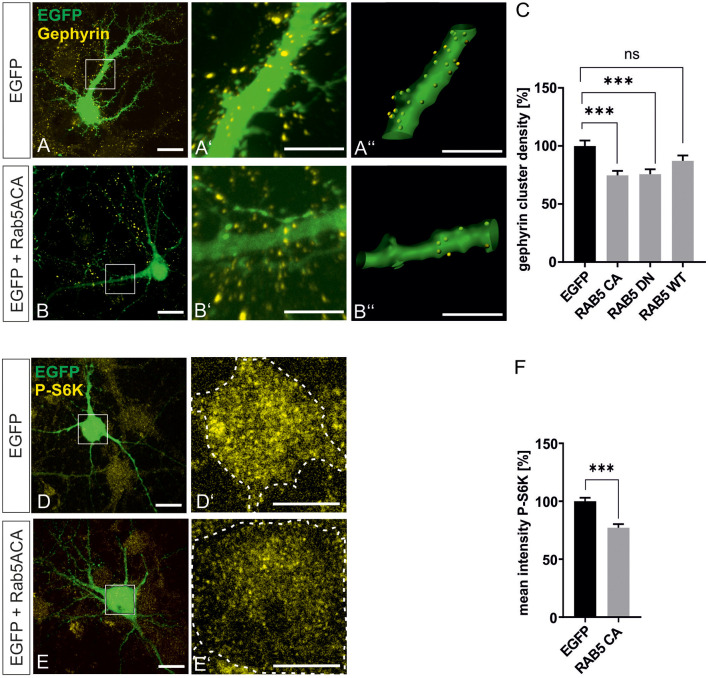
Gephyrin clustering relies on endosomal functions. Hippocampal neurons were transfected with EGFP or rab5-EGFP expression plasmids. Representative images depict EGFP fluorescence and gephyrin staining shown for EGFP control **(A–A”)** or rab5A-CA **(B–B”)**. Overview images are shown in panels (**A,B**; scale bar 20 μm) while insets are enlarged in **(A’,A”,B’,B”)** (scale bar 10 μm). Panels **(A’,B’)** depict confocal images,** (A”,B”)** processed images for the quantification of gephyrin cluster densities within masks of EGFP-positive dendritic segments (25 μm). **(C)** Quantification of gephyrin cluster densities. Kruskal–Wallis test *H*_(3)_ = 19.11. EGFP: *n* = 62, RAB5A-CA *n* = 63, RAB5A-DN *n* = 57, RAB5A-WT *n* = 58. ***p < 0.0001, error bars SEM. **(D,E)** EGFP **(D)** or RAB5A-CA transfected **(E)** hippocampal neurons were stained for phosphorylated p70S6K (pS6K, scale bar 20 μm). Insets are enlarged in (**D’,E’**; scale bar 10 μm) for determination of mean fluorescence intensities of pS6K signals within a mask of EGFP-positive cell bodies. Dashed outlines mark the area of the cell bodies. **(F)** Quantification of pS6K intensities. EGFP, *n* = 39; RAB5A-CA, *n* = 35. *t*-test. ***p < 0.0001, error bars: SEM. ns, not significant.

mTOR becomes activated in the late endosomal compartment after interaction with active Rheb-GTP (Flinn et al., 2010; Bar-Peled and Sabatini, 2014). For further support of our finding that mTOR signaling is involved in gephyrin clustering, we interfered with early-to-late endosome conversion to achieve inhibition of mTOR signaling in hippocampal neurons essentially as shown in a previous report (Flinn et al., 2010). Rab5A wild type (Rab5A wt), dominant-negative Rab5A (Rab5A DN), and constitutively active Rab5A (Rab5A CA) expression plasmids were transfected into hippocampal neurons (Figure 7). As observed in other cell types, mTOR activation was impaired by Rab5A CA expression as shown by reduced S6K phosphorylation (Figures 7D–F). We then quantified gephyrin cluster densities after transfection of RabA 5 expression plasmids (Figures 5A–C). Both Rab5A CA and Rab5A DN decreased gephyrin clustering. Transfection of WT, DN, or CA expression plasmids for Rab4, 7, 8, and 11 did not modulate gephyrin clustering indicating that the function of late endosome-to-lysosome conversion or of recycling endosomes are dispensable for gephyrin clustering (Supplementary Figure 3). It is of note that an earlier report showed that interference with Rab 7 and 11 did not disturb mTOR activation, which is completely in line with our result that mTOR-dependent gephyrin clustering remained unaffected (Flinn et al., 2010). In conclusion, the results further support the hypothesis that mTOR activation in the late endosomal compartment contributes to the control of gephyrin clustering.

### Tsc2 Regulates Gephyrin Clustering

Given the central role of mTOR for the stabilization of inhibitory synapses dependent on Met signaling, it is conceivable that Tsc2, another autism risk gene and negative regulator of mTOR, might also be involved in gephyrin clustering. Two independent lentiviral knockdown constructs showed reduced Tsc2 expression at the mRNA and the protein level after lentiviral miRNA expression in primary hippocampal neurons (Joly et al., submitted).

**Figure 8:**
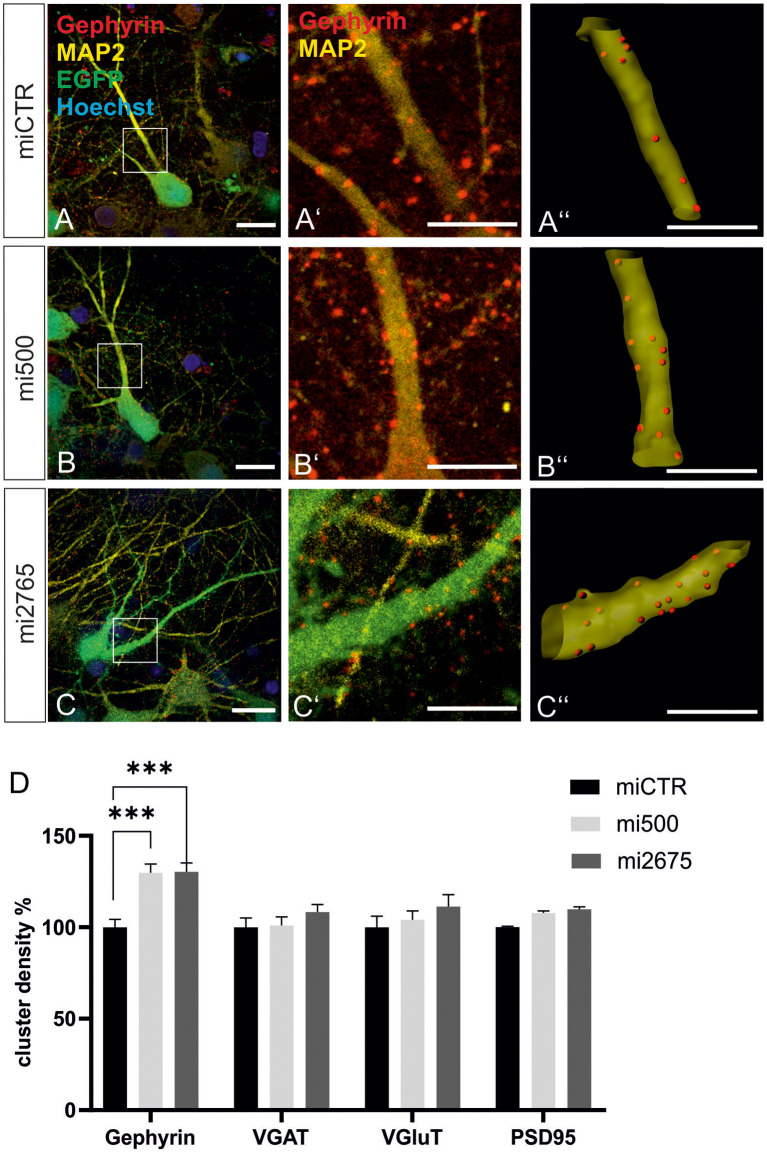
Tsc2 knockdown increases gephyrin clustering *in vitro*. **(A–C)** Hippocampal neurons were transduced with lentiviral vectors expressing miCTR **(A)**, or the Tsc2 knockdown constructs mi500 **(B)** or mi2675 **(C)**. Scale bar 20 μm. Insets in panels **(A–C)** are enlarged in the corresponding images **(A’–C’,A”–C”)**. Scale bar 10 μm. Panels **(A’–C’)** show confocal images depicting gephyrin (red) and the neuronal marker MAP2 (yellow). **(A”–C”)** Gephyrin spots detected on MAP2-positive dendritic segments used as a mask for the identification of gephyrin spots. **(D)** Quantification of synaptic marker densities on dendritic segments of 25 μm in length. Two-way ANOVA and Tukey’s *post hoc* test *F*_(2,657)_ = 9.780. Gephyrin: miCTR *n* = 114, mi500 *n* = 75, mi2675 *n* = 75. VGaT: miCTR *n* = 45, mi500 *n* = 44, mi2675 = 44. PSD95: miCTR *n* = 45, mi500 *n* = 46, mi2675 *n* = 45. VGluT: miCTR *n* = 46, mi500 *n* = 45, mi2675 *n* = 45. ***p < 0.0001. Error bars: SEM.

We then studied the impact of Tsc2 knockdown on gephyrin clustering in hippocampal neurons *in vitro*. Quantitative immunocytochemistry of synaptic marker proteins revealed significantly increased gephyrin cluster densities after transduction with Tsc2 knockdown vectors (Figures 8A–C”, quantified in **D**). These results are in line with the hypothesis that growth factor signaling activates mTOR signaling through activation of the PI3K-Akt pathway and concomitant inactivation of Tsc2. The presynaptic marker protein VGAT remained unaffected as observed for the glutamatergic synapse markers VGLUT and PSD95. The experiment confirms a link between mTOR activation and gephyrin clustering.

## Discussion

In summary, our results imply a function of Met in the regulation of gephyrin clustering through mTOR signaling. *In vivo* and *in vitro* experiments suggest an activation of the canonical mTOR pathway through the inactivation of Tsc1/2. For Tsc2 knockdown, we observed increased gephyrin clustering *in vitro* implying a link between gephyrin clustering, growth factor signaling, and PI3K-Akt-mTOR activation. It is conceivable that synaptic transmission is modulated by an interplay between morphological and functional alterations potentially inducing E/I imbalance in the neuronal network. Our report clarifies the role of inhibitory synapse stabilization by the ASD risk genes Met and Tsc2 at the morphological level.

Gephyrin clustering is controlled by growth factor signaling and the activation of the PI3K-Akt-mTOR pathway (Sabatini et al., 1999; Kneussel and Betz, 2000; Wuchter et al., 2012; Beuter et al., 2016). Gephyrin clustering by itself is a crucial step for the stabilization of inhibitory synapses (Kneussel et al., 1999). We now show that the previously suggested reduction in GABAergic transmission after inhibition of Met expression correlates with reduced gephyrin clustering and a loss of VGAT-positive, presynaptic GABAergic terminals (Lo et al., 2016). Since PSD95 and VGLUT marker densities for excitatory synapses remained unaffected, E/I imbalance may be linked to Met expression and the reduction of GABAergic innervation at least at the morphological level. It is of note that our RNA interference experiment considers synaptic alterations in injected juvenile animals. Therefore, we primarily observed the destabilization of preformed inhibitory synapses in the absence of developmental mechanisms. However, the *in vivo* results here are fully compliant with the *in vitro* results obtained in hippocampal neurons as an approximation for a more immature, developmental phenotype. CamKII promoter-driven lentiviral miRNA expression confines Met knockdown to postsynaptic compartments of principal neurons receiving GABAergic presynaptic input from interneurons. In contrast, a previous observation defines presynaptic Met to control synapse formation through interaction with postsynaptic Sema4D (Frias et al., 2019). However, Met expression has also been observed in dendrites and dendritic spines suggesting expression in postsynaptic compartments (Eagleson et al., 2013).

In a series of previous reports, we have shown that receptor tyrosine kinases including Fgfr1, trkB, and EphA7 are involved in the control of gephyrin clustering (Kriebel et al., 2011; Wuchter et al., 2012; Beuter et al., 2016). Here, we add Met as a further receptor tyrosine kinase, which shares the property to engage mTOR signaling for gephyrin clustering. However, in the case of Fgfr1, its association with neurofascin and the restricted expression of neurofascin at the axon initial segment confines the stabilization of GABAergic synapses to axo-axonic synapses. For EphA7, the expression on proximal dendritic segments and on somata allows for the control of basket cells (Beuter et al., 2016). Whether the action of Met with regard to gephyrin clustering is locally restricted remains to be elucidated. However, colocalization of Met with gephyrin in all parts of dendritic segments and the soma of granule cells argues against a role in specific compartments.

We further showed that Met controls gephyrin clustering in hippocampal neurons through mTOR. Our working hypothesis is supported by several arguments. First, HGF activated Met and downstream mTOR for gephyrin clustering which was sensitive to the mTOR inhibitor rapamycin. Second, Tsc2 down-regulation and concomitant activation of mTOR induced gephyrin clustering in hippocampal neurons. Third, inhibition of mTOR signaling through the perturbation of endosomal mTOR activation ended up with reduced gephyrin clustering. Altogether, our results imply that Met-dependent gephyrin clustering is governed by the PI3K-Akt signaling pathway. In this line, a kinome-wide siRNA screen for genes involved in gephyrin clustering identified a set of receptor tyrosine kinases and the corresponding downstream signaling components including AKT and MAPK (Wuchter et al., 2012). We did not evaluate the contribution of MAPK signaling to HGF-induced gephyrin clustering. It is, however, of note that the siRNA screen also identified the ASD risk gene Dyrk1A which may control MAPK signaling through sprouty (Evers et al., 2017; Dang et al., 2018; Raveau et al., 2018). It is therefore tempting to speculate that known ASD risk genes including Met, Pten, Tsc2, Rheb as well as Dyrk1A affect MAPK and PI3K-Akt pathways, converge on mTOR, and altogether are implicated in the control of gephyrin clustering.

Gephyrin clustering relies on the GTPase Rab5A expressed on early endosomes, which is involved in early/late endosome conversion as well as activation of mTOR in the late endosomal compartment (Flinn et al., 2010). mTOR becomes recruited to Rheb-GTP at late endosomes which is a prerequisite for its activation in the presence of amino acids and growth factors (Bar-Peled and Sabatini, 2014). In accordance with previous reports (Flinn et al., 2010), we find decreased mTOR activity in neurons upon overexpression of Rab5A CA as measured by decreased p70S6K phosphorylation. This is in accordance with our finding that gephyrin clustering relies on mTOR activation. It is of interest in this context that we have previously identified PIK3C3 as a crucial protein kinase for gephyrin clustering in a genome-wide siRNA screen (Wuchter et al., 2012). PI3KC3 interacts with and becomes activated by early endosome Rab5A and late endosome Rab7 (Murray et al., 2002; Stein et al., 2003). PI3KC3 gives yield to phosphatidylinositol 3-phosphate which is important for the assembly of gephyrin clusters (Papadopoulos et al., 2017). Requirement of both early/late endosome conversion and mTOR activation let therefore suggest a role for the late endosomal compartment for the control of gephyrin clustering.

In conclusion, our results elucidate the impact of Met and mTOR-related downstream signaling on the stabilization of GABAergic synapses. Our results also integrate Met and Tsc2 into a joint ASD disease pathway.

## Data Availability Statement

The original contributions presented in the study are included in the article/Supplementary Material, further inquiries can be directed to the corresponding author.

## Ethics Statement

The animal study was reviewed and approved by Regierungspräsidium Tübingen.

## Author Contributions

MK constructed and validated Tsc2 and Met knockdown vectors. All other experiments were conducted by PJ. HV designed the study and supervised the experiments as well as wrote the manuscript. All authors contributed to the article and approved the submitted version.

## Conflict of Interest

The authors declare that the research was conducted in the absence of any commercial or financial relationships that could be construed as a potential conflict of interest.
