# Supplementary material for: Autism Spectrum Disorder Risk Factor Met Regulates the Organization of Inhibitory Synapses
Source: Front Mol Neurosci. 2021 May 13;14:659856. doi: 10.3389/fnmol.2021.659856 (PMC8155383; doi:10.3389/fnmol.2021.659856)
Supplement: Supplementary file 1 [file Data_Sheet_1.pdf]

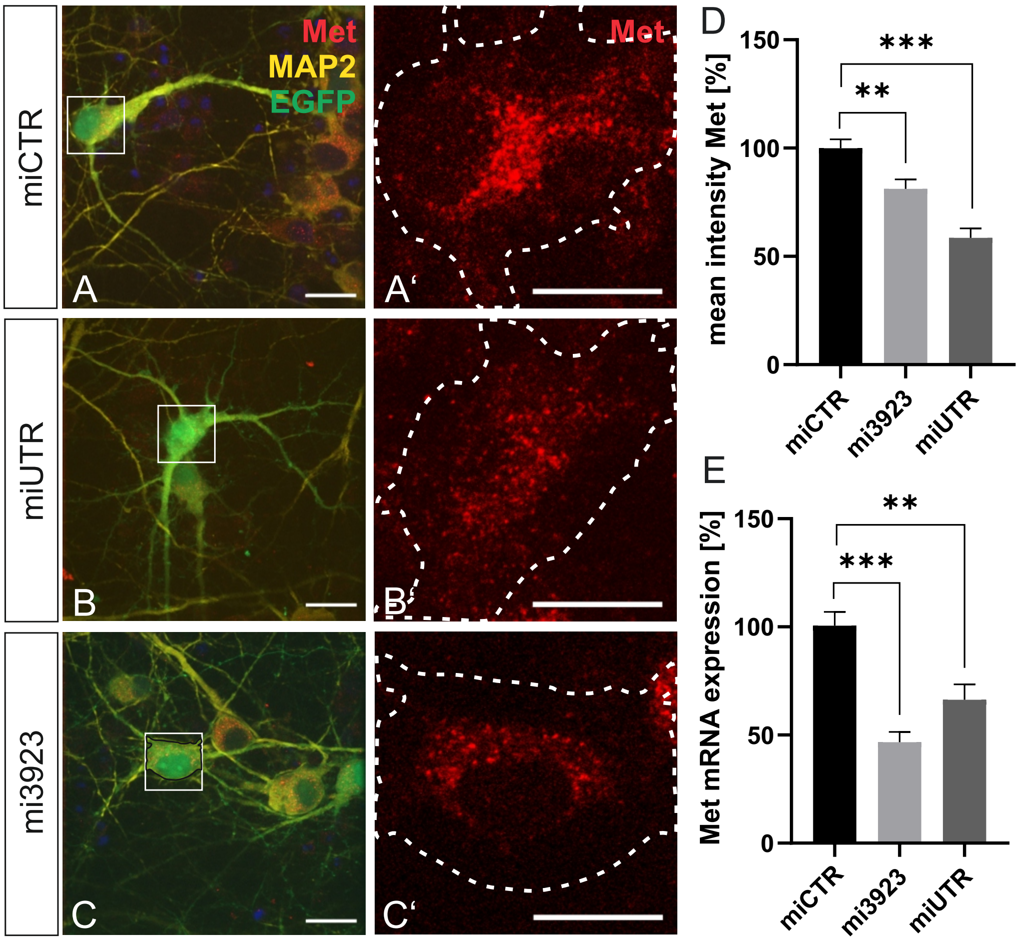

Suppl Fig S1

Met knockdown *in vitro*. A-C: Hippocampal neurons were infected with lentiviral vectors expressing EGFP for control of infection as well as a control miRNA (miCTR, A), and miRNA specific for Met (mi3923, B; miUTR, C). Insets in A-C are magnified in A'-C' (scale bars A-C 20 µm, A'-C' 10 µm). Dashed outlines mark the area of the cell bodies. D: MAP2-positive cell bodies were used as a mask to quantify mean intensity of immunofluorscent Met signals. One-way ANOVA and Dunnett's test; $F(2,191)=4.984$; ** $p=0.0065$, *** $p<0.0001$. miCTR: n=103; mi3923: n=50; miUTR: n=42. Error bars s.e.m. (E) qRT-PCR of cortical neurons infected with lentiviral vectors expressing miCTR, mi3923, and miUTR. One-way ANOVA and Dunnett's test; $F(2,43)=0.5996$, ** $p=0.0019$, *** $p<0.0001$. miCTR: n=22; mi3923: n=14; miUTR: n=10. Error bars s.e.m.
