# Supplementary material for: Autism Spectrum Disorder Risk Factor Met Regulates the Organization of Inhibitory Synapses
Source: Front Mol Neurosci. 2021 May 13;14:659856. doi: 10.3389/fnmol.2021.659856 (PMC8155383; doi:10.3389/fnmol.2021.659856)
Supplement: Supplementary file 2 [file Data_Sheet_2.pdf]

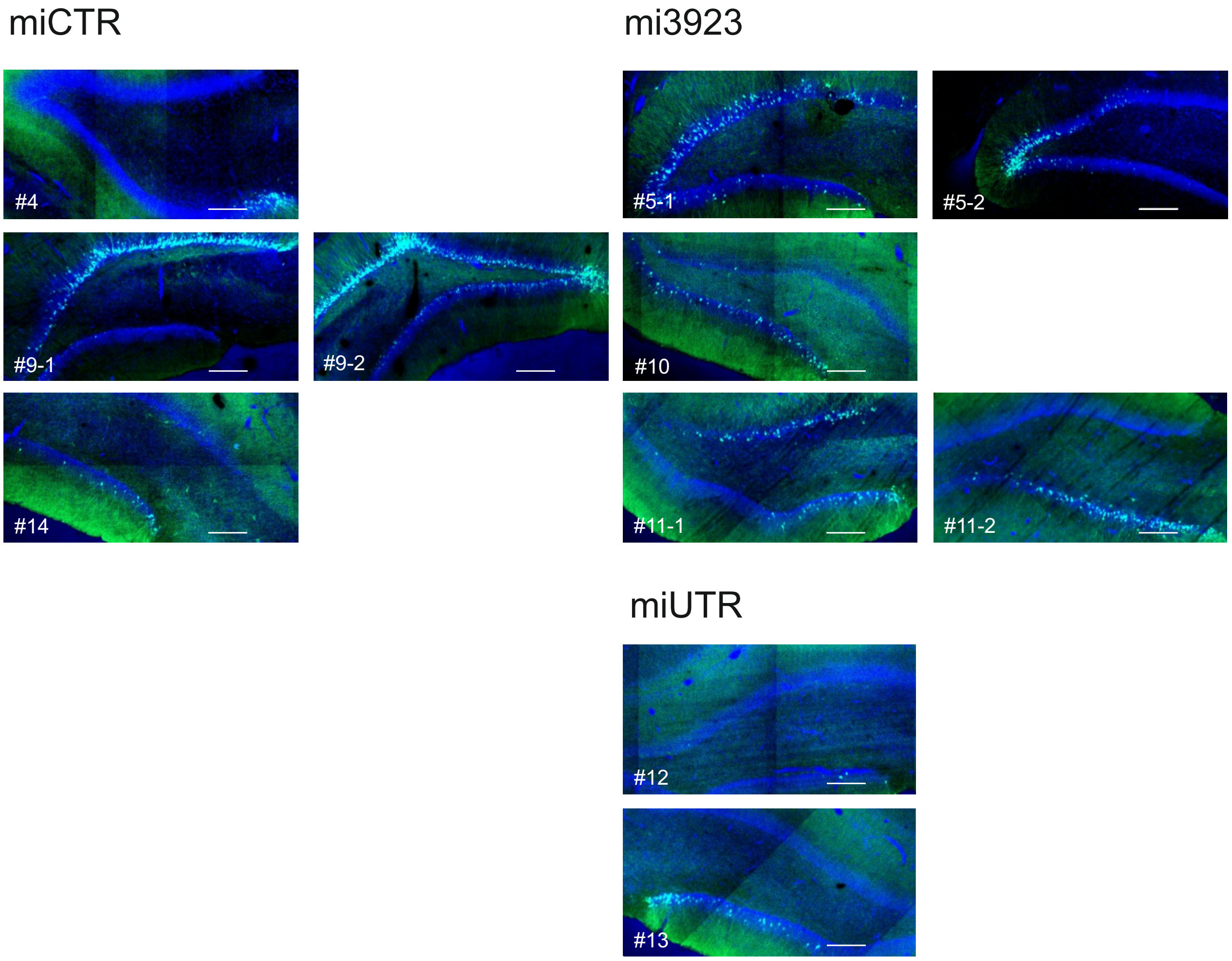

Suppl. fig. S2. Injection sites of lentiviral vectors miCTR, mi3923, and miUTR in the dentate gyrus of rats. Injection sites were visualized by coexpressed EGFP while nuclei of granule cells are stainded by Hoechst. Numbers depict individual animals while extensions indicate contralateral hemispheres of the same animal.
