# Supplementary material for: Autism Spectrum Disorder Risk Factor Met Regulates the Organization of Inhibitory Synapses
Source: Front Mol Neurosci. 2021 May 13;14:659856. doi: 10.3389/fnmol.2021.659856 (PMC8155383; doi:10.3389/fnmol.2021.659856)
Supplement: Supplementary file 3 [file Data_Sheet_3.pdf]

A

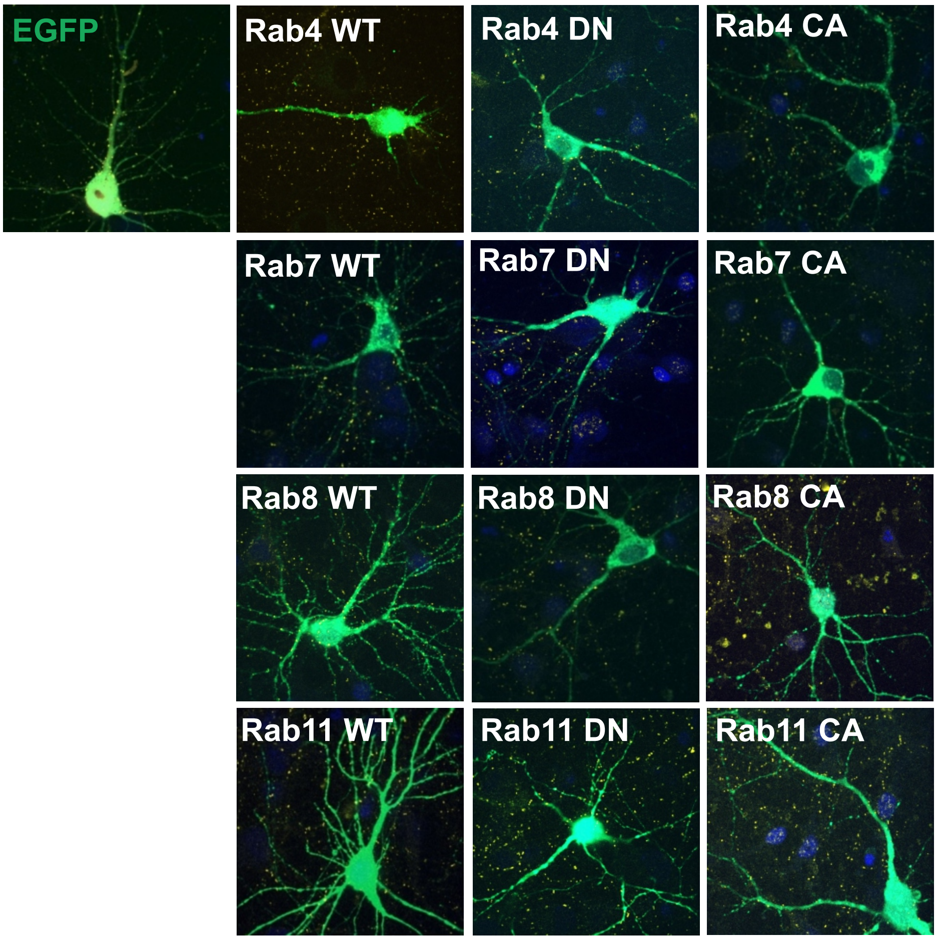

B

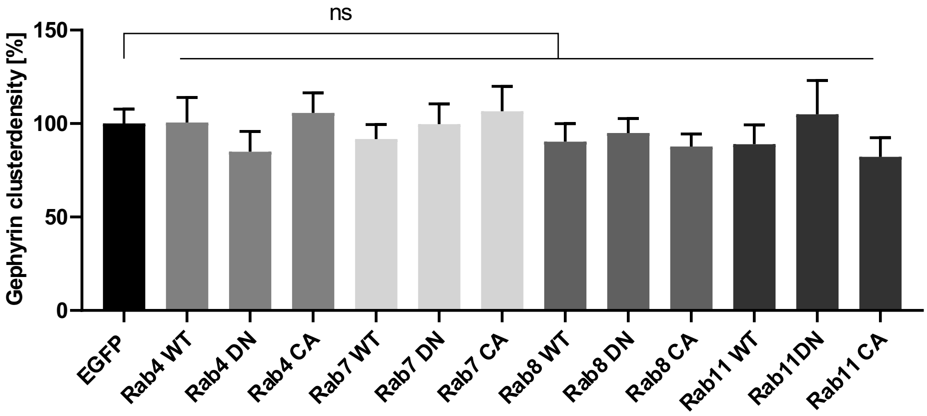

Suppl fig S3: (A) Cotransfection of Rab variants Rab4,7,8, 11 (each as wildtype, dominant-negative or constitutively active mutant) with a EGFP expression vector. (B) Gephyrin cluster densities were quantified on EGFP-positive neurites. Kriskal-Wallis test H(12)=8.570. ns: not significant. EGFP (control) n= 48; Rab4WT n=20; Rab4DN n=12; Rab4CA n=20; Rab7WT n=33; Rab7DN n=27; Rab7CA n=24; Rab8WT n=29; Rab8DN n=21; Rab8CA n=29; Rab11WT n=40; Rab11DN n=11; Rab11CA n=26; error bars s.e.m.
